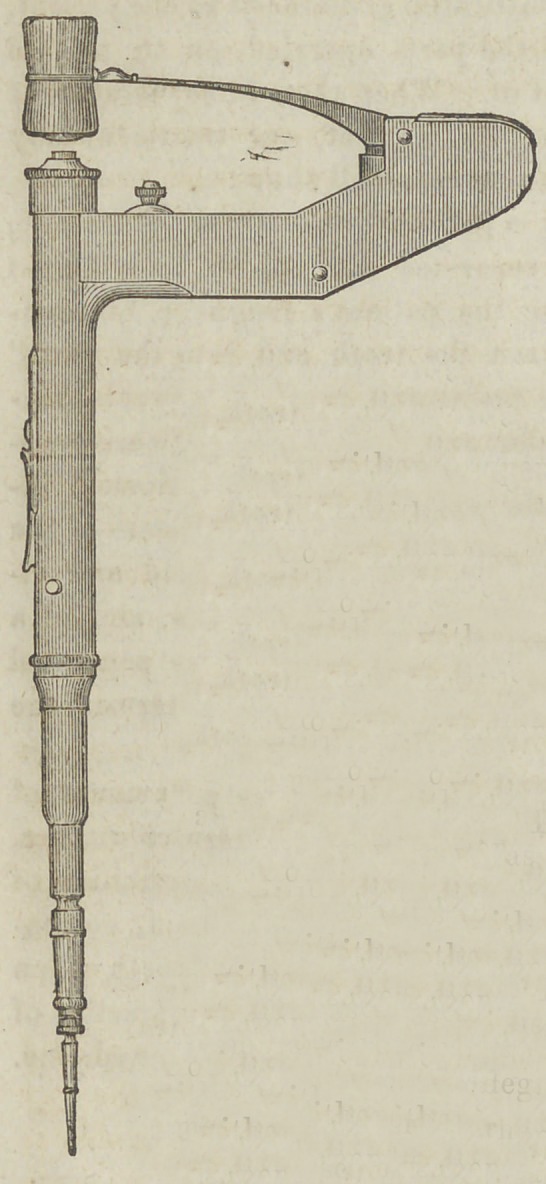# Redman’s Mallet Plugger

**Published:** 1867-01

**Authors:** 


### Editorial.

### REDMAN'S MALLET PLUGGER.

The accompanying illustration gives an idea of this very ingenious, and useful instrument; it presents little or nothing, however, of its interior structure. This we cannot pretend here to describe, of which suffice it to say, that it is of such a character as to operate definitely and accurately, and that too without a liability of getting out of repair. The adjustments are such as not to be readily displaced or broken.

This instrument comes nearer our idea of what the automatic mallet should be, than any we have seen; in this, we have more particular reference to the character of the stroke. The principle of operating the instrument is the same as that of Snow, Scranton, Salmon, and others. The blow is made by a veritable mallet, which gives a spring stroke, and can be regulated to any degree of force between the lightest, and heaviest ever required. The large arm attached would seem to make it unwieldy; but use of, and familiarity with the instrument will soon, chiefly, if not altogether remove that objection. We have used the plugger somewhat for the past two months, and like it very much as a condenser; though as in the case of other automatic mallets, we have found a few patients who object more to its stroke than to the mallet in the hand of the assistant. This instrument has a stop to its movement, that enables it to be used by hand pressure. The weight of the instrument renders it less practicable for taking up and introducing the gold, than some others.

It will necessarily be somewhat expensive, but we think after some familiarity, it would be almost invaluable in the hands of any one. Further information concerning it can be obtained by addressing Dr. W. G. Redman, Louisville, Ky.

## Figures and Tables

**Figure f1:**